# Ipsilesional volume loss of basal ganglia and thalamus is associated with poor hand function after ischemic perinatal stroke

**DOI:** 10.1186/s12883-022-02550-3

**Published:** 2022-01-12

**Authors:** Nigul Ilves, Silva Lõo, Norman Ilves, Rael Laugesaar, Dagmar Loorits, Pille Kool, Tiina Talvik, Pilvi Ilves

**Affiliations:** 1 Radiology Clinic, Tartu University Hospital, Tartu, Estonia; 2 Department of Radiology, University of Tartu, L. Puusepa 8, 51014 Tartu, Estonia; 3 Department of Pediatric Neurology, University of Helsinki; Helsinki University Hospital, Helsinki, Finland; 4 Department of Pediatrics, University of Tartu, Tartu, Estonia; 5 Children’s Clinic, Tartu University Hospital, Tartu, Estonia

**Keywords:** Perinatal stroke, Basal ganglia, Thalamus, MRI, Volumetrics, Motor outcome

## Abstract

**Background:**

Perinatal stroke (PS) is the leading cause of hemiparetic cerebral palsy (CP). Involvement of the corticospinal tract on neonatal magnetic resonance imaging (MRI) is predictive of motor outcome in patients with hemiparetic CP. However, early MRI is not available in patients with delayed presentation of PS and prediction of hemiparesis severity remains a challenge.

**Aims:**

To evaluate the volumes of the basal ganglia, amygdala, thalamus, and hippocampus following perinatal ischemic stroke in relation to hand motor function in children with a history of PS and to compare the volumes of subcortical structures in children with PS and in healthy controls.

**Methods:**

Term born PS children with arterial ischemic stroke (AIS) (*n* = 16) and with periventricular venous infarction (PVI) (*n* = 18) were recruited from the Estonian Pediatric Stroke Database. MRI was accuired during childhood (4-18 years) and the volumes of the basal ganglia, thalamus, amygdala and hippocampus were calculated. The results of stroke patients were compared to the results of 42 age- and sex-matched healthy controls. Affected hand function was evaluated by Assisting Hand Assessment (AHA) and classified by the Manual Ability Classification System (MACS).

**Results:**

Compared to the control group, children with AIS had smaller volumes of the ipsi- and contralesional thalami, ipsilesional globus pallidus, nucleus accumbens and hippocampus (*p* < 0.005). Affected hand function in children with AIS was correlated with smaller ipsilesional thalamus, putamen, globus pallidus, hippocampus, amygdala and contralesional amygdala (r > 0.5; *p* < 0.05) and larger volume of the contralesional putamen and hippocampus (r < − 0.5; *p* < 0.05).

In children with PVI, size of the ipsilesional caudate nucleus, globus pallidus, thalamus (*p* ≤ 0.001) and hippocampus (*p* < 0.03) was smaller compared to controls. Smaller volume of the ipsi- and contralesional thalami and ipsilesional caudate nucleus was correlated with affected hand function (r > 0.55; *p* < 0.05) in children with PVI.

**Conclusions:**

Smaller volume of ipsilesional thalamus was associated with poor affected hand function regardless of the perinatal stroke subtype. The pattern of correlation between hand function and volume differences in the other subcortical structures varied between children with PVI and AIS. Evaluation of subcortical structures is important in predicting motor outcome following perinatal stroke.

## Introduction

Perinatal stroke (PS) is an increasingly acknowledged cause of significant lifelong motor impairment, but causes also combined neurocognitive morbidity [1–3]. PS is defined to occur from 20 weeks of fetal life through the 28th day of postnatal life secondary to arterial or venous thrombosis or embolization [4]. Two vascular types of injury, arterial ischemic stroke (AIS) and periventricular venous infarction (PVI), predominate among PS syndromes [2, 5, 6]. Some children with PS are asymptomatic at birth [5] and appear to need medical attention for asymmetric motor development or developmental delay later in infancy [1]. Radiologic imaging shows chronic changes after stroke, which are presumed to occur during the pre- or perinatal period. Neonatal and presumed perinatal AIS are caused by large artery occlusions, usually in the middle cerebral artery (MCA) territory, with cortical and subcortical injuries, most often acquired near term birth. The established mechanism of PVI in preterm infants is germinal matrix hemorrhage before 34 weeks of gestation, leading to compression of the medullary veins and focal venous infarction of the periventricular white matter. Presumed PVI in term born children is thought to represent an in-utero version of the same stroke [5, 7–9].

AIS in proximal MCA leads to infarction of both the distal hemispheric tissue and basal ganglia (sparing globus pallidus and caudate head), while occlusion in the distal division is assumed to spare the basal ganglia. In rare cases stroke occurs only in lateral lenticulostriate arteries, infarcting the basal ganglia (putamen and caudate body) and the posterior limb of the internal capsule (PLIC), sparing the subcortical white matter and cortex [7, 10]. Periventricular venous infarction causes porencephalic enlargement of the side ventricle and/or periventricular porencephalic cysts [7]. What is common to all PS is damage to the developing sensorimotor system, including the motor and somatosensory cortices and the corticospinal tracts, which results in contralesional spastic hemiplegia [1, 8, 11, 12]. Motor impairment has been found to depend on the relative volume of lesion on acute diffusion imaging [13] and on combined damage to various sensorimotor tracts [14].

Early prediction of unilateral motor impairment in children with AIS has been associated with involvement of the main MCA branch and PLIC, or with concomitant injury to the hemispheric tissue, basal ganglia and PLIC, established by neonatal magnetic resonance imaging (MRI), while injury to only one or two of these sites is associated with good outcome [15–18]. Absence of involvement of the corticospinal tract has resulted in normal motor development [19]. However, early structural asymmetries of the corticospinal tract can not always predict presence and, first and foremost, severity of later hemiparesis [20]. In some follow-up studies, children with neonatal AIS sparing the primary motor system (i.e. precentral gyrus, basal ganglia/thalamus, internal capsule) have not been found to develop cerebral palsy (CP) [19, 21].

The major impact of basal ganglia damage on hand function has been shown in several studies [22–24], but there is scanty information about comparison of vascular subtypes of ischemic perinatal stroke.

Children with presumed PS with delayed diagnosis do not have acute stage images and the prognosis of eventual motor outcome remains a challenge. It is unknown if the same topographic predictors of adverse motor outcome applicable in AIS can also be applied in presumed PVI in term infants. In our recent study, large stroke involving several lobes of the brain predicted poorer overall neurological outcome in children with AIS, but not PVI [1].

Reorganization of the motor system has been shown to take place after perinatal unilateral brain injury [25–27]. Predicting reorganization patterns and guiding/inducing plasticity could help improve outcomes. It is still poorly understood how key sensorimotor networks and structures remote from injury are disrupted after early stroke. Some studies have reported thalamic diaschisis in adult stroke [28, 29]. A diffusion imaging study of pediatric stroke has found that the thalamus is the most common location of acute diaschisis [30]. While typical PS lesions do not cause direct damage to the cerebellum and thalamus, their high connectivity to injured areas may result in structural and functional alterations [31]. Following PS, ipsilesional thalamic volume was reduced in both AIS and PVI, but this was not associated with clinical motor function [12]. Contralesional hand performance after neonatal AIS has been shown to correlate with atrophy in brain areas directly or functionally connected to infarct, but anatomically remote from it, such as mediodorsal thalamus and cerebellum [32].

The aim of this study was to evaluate the volumes of the ipsi- and contralesional basal ganglia, amygdala, thalamus, and hippocampus following perinatal ischemic stroke in relation to hand motor function in children with a history of perinatal AIS and PVI and to compare the volumes of subcortical structures in children with perinatal stroke and in healthy controls.

## Material and methods

### Patients

This is a population based consecutive cohort study, which is part of a larger study on outcome of perinatal stroke [1, 5, 33].

The participants were identified from the population based Estonian Pediatric Stroke Database (PSD) containing the data of children with PS collected during an epidemiological study retrospectively from 1994 and prospectively from 2004 [34]. Before the inclusion of patients in PSD, two pediatric neuroradiologists, who were blinded to clinical findings, independently reviewed radiological images and classified PS according to the vascular type of stroke [7]. Term or near term born children with a gestational age of at least 36 weeks were included in PSD if they fulfilled one of the following criteria: (1) children with neonatal AIS in the MCA territory, diagnosed with acute ischemic stroke between birth and 28 days of life on the basis of neonatal MRI; (2) children with presumed AIS diagnosed after 28 days of life in the MCA territory with chronic changes after stroke on MRI; (3) term born children with presumed PVI diagnosed after 28 days of life with MRI revealing a lesion consistent with remote periventricular venous infarction. The exclusion criteria for PSD were the following: (1) documented diseases other than stroke involving the central nervous system (e.g. severe birth asphyxia, watershed infarction, kernicterus, encephalitis, hypoglycemia with watershed bilateral infarction, mitochondrial disease and tumor), (2) other cortical malformation or congenital anomaly, and (3) absence of confirmative cranial imaging (MRI).

Children were included in the study if they fulfilled all the criteria: 1) record in the Estonian PSD with confirmed diagnosis of neonatal AIS, presumed AIS or presumed PVI; 2) birth after 36 gestational weeks; 3) informed consent to participate in the follow-up study; 4) follow-up 3 T MRI scan at age 6–18 years performed for scientific purposes without anesthesia; 5) affected hand function measured by the Assisting Hand Assessment (AHA) test between 3.5 and 13 years of age.

The control group of healthy volunteers consisted of age and sex matched children (*n* = 42) aged 8–18 years. The control group was recruited from among the coworkers’ relatives and children and expanded by involving their friends and classmates.

### Data abstraction

#### Method for MRI acquisition and processing

A follow-up MRI was acquired specifically for the current study after the acute stage of PS at the age of 6–18 years, with a 3 T Philips Achieva scanner using a 8-channel SENSE head coil 3.0 T/8ch (Philips Medical Systems, Best, The Netherlands). Structural T1-weighted images were obtained using a fast field echo sequence, TR = 8.2 ms TE = 3.8 ms. The field of view was 256 × 256 mm and the voxels were isotropic, 1 × 1 × 1 mm.

Analysis was performed, using the FMRIB Software Library (FSL) (https://www.fmrib.ox.ac.uk/fsl/) version 6.0.2. Raw images were converted to the NIFTI format and anonymized by an investigator (Ni.I) who was blinded to clinical data. The images of right-side lesions were flipped along the x-axis so that all the lesions were analyzed as left-side lesions.

We used two methods for analysing imaging data: voxel based morphometry and volumetric analysis by segmentation.

#### Voxel based morphometry

FSL-VBM [35], (http://fsl.fmrib.ox.ac.uk/fsl/fslwiki/FSLVBM), an automated optimised voxel based morphometry (VBM) protocol [36], was used unaltered, with quality control after each processing step. Structural images were brain-extracted and gray matter-segmented and registered to the Montreal Neurological Institute (MNI 152, Montreal Neurological Institute, Montreal, QC, Canada) standard space using non-linear registration.

The left-right symmetric study-specific gray matter template was generated by averaging and flipping images along the x-axis. To eliminate any bias due to varying group size, a random sample with equal group size was formed of the PVI, AIS and control groups for template generation.

All initial gray matter images were non-linearly registered to the study-specific template and “modulated” to correct for local expansion (or contraction) due to the non-linear component of spatial transformation. The modulated gray matter images were then smoothed with an isotropic Gaussian kernel with a sigma of 4 mm.

A voxelwise general linear model comparing the control, PVI and AIS groups (all 6 pairwise comparisons), with age at the time of MRI as a confounder, was applied by using permutation-based non-parametric testing with 5000 steps, correcting for multiple comparisons across the space. The affected brain regions were identified using the FSL atlasquery tool and the Harvard-Oxford Subcortical Structural Atlas.

#### Volumetric analysis by segmentation

The structures of the subcortical gray matter, i.e. the thalamus, caudate nucleus, putamen, globus pallidus, hippocampus, amygdala and nucleus accumbens, were automatically segmented using the FSL FIRST tool [37] in both brain hemispheres. After quality control, the segmentation faults caused by stroke-induced morphologic changes were corrected by excluding the lesion areas and by manually correcting segmentation, using the FSL tool FSLeyes (by researcher Ni. I and extended separately by radiology resident No. I). After 2 weeks, quality control and correction were repeated in a randomized subject order, with the evaluators blinded to the group type and clinical outcome. The FSL tool Fslstats was used to measure the volume of segmentations.

The segmentation volumes were normalized using an individual volumetric *scaling factor* generated by the FSL’s SIENAX tool for converting the subject’s brain volume to MNI152 standard space volume [38]. The scaling factor is a measure for individual’s head size.

#### Evaluation of affected hand function

The global neurodevelopmental outcome of the patients was recently described [1] and evaluated using the Pediatric Stroke Outcome Measure (PSOM) [39]. PSOM contains five subscales: right sensorimotor, left sensorimotor, language production, language comprehension, and cognitive/behavioral performance. Each subscale yields a deficit severity score: 0 (no deficit), 0.5 (mild but no impact on function), 1 (moderate with some functional limitations), and 2 (severe with missing function). The neurological motor outcome was evaluated by a pediatric neurologist (R.L or S.L). Moderate to severe unilateral sensorimotor deficit correlated with definition of congenital spastic hemiparesis (e.g. unilateral spastic cerebral palsy): unilateral increased muscle tone and pathological reflexes, resulting in an abnormal pattern of movement and posture [40].

The function of the affected hand was evaluated, according to the Assisting Hand Assessment (AHA) version 4.4 [41], by a pediatric neurologist (SL) who was blinded to the neuro-imaging data. AHA was administered during overall outcome evaluation at pre-school to school age when children had reached appropriate age for bimanual hand function assessment.

AHA is a Rasch analysis-based performance scale that measures how effectively children with unilateral impairment spontaneously use their affected hand during bimanual activities. Different test items describing various object-related hand actions are scored on a 4-point scale rating the quality of performance. Raw scores were converted, using Rasch analysis, to logit scores varying between 0 and 100, with higher scores indicating higher ability. Bimanual function was classified according to the Manual Ability Classification System (MACS), which describes how children with CP use their hands to handle objects during daily activities. Five levels are based on the children’s self-initiated ability to handle objects and on their need for assistance or adaptation to perform manual activities in everyday life. Children at level I handle objects easily and successfully with possible limitation in the ease of performing manual tasks requiring speed and accuracy. Children at level II handle most objects but with somewhat reduced quality and /or speed of achievement. At level III, objects are handled with difficulty and children need help to prepare for and/or modify activities. At level IV, children handle a limited selection of easily managed objects in adapted situations, and at level V children can not handle objects and need full assistance [42].

The reasearchers performing the volumetric analysis of anonymized MRI images were blinded to the clinical evaluation performed by child neurologists. The data of radiological and clinical evaluation was analyzed by a statistician who combined both datasets.

### Statistical analysis

Statistical analysis was made with the SAS Version 9.4 (SAS Institute INC, Cary, NC) statistical software and the statistical package R version 3.6.2. Correlations between AHA units and volumes of the subcortical structures were calculated. Spearman’s rank coefficients were used where applicable. Shapiro-Wilk test was used for assessment of normality. Continuous variables are presented as mean values with the 95% confidence intervals (CI) or as median values with the interquartile range (IQR), while qualitative variables are presented as absolute and relative frequencies. Statistical comparisons between normally distributed continuous variables and categorical variables were performed by one-way analysis of variance (ANOVA) using Levene’s test to compare variances. In the case of asymmetric continuous variables, the tested hypotheses were based on the calculations of a nonparametric test such as Kruskal-Wallis test. Multiple comparisons were made if a significant difference across the groups was noted. For each pairwise comparison of groups, Student’s *t* test or Wilcoxon-Mann-Whitney test was used as appropriate. To compare proportions (qualitative variables), χ^2^ test, Fisher’s exact test (when the expected frequency was lower than 5) were used. Corrections for multiple testing were made using the false discovery rate (FDR) linear step-up procedure [43]. Benjamini-Hochberg critical values were calculated as (i/m)Q, where i is the rank in an ascending list of *p* values, m is the total number of tests, and Q is an FDR. Only raw p values that are below the adjusted FDR significance threshold are therefore significant and marked as such in bold in the tables. Multiple FDR significance levels were used, 0.05 for subcortical volumes and 0.08 for hand function correlations, because we did not want to set the proportion of false negatives too high (for the risk of missing a potentially important finding) [44, 45]. All raw *p*-values shown are two-tailed.

## Results

### Population

For this study, 42 PS patients were initially included; eight children without AHA testing or with artefacts on MRI were excluded. The final group of PS consisted of 34 patients, 7 children had neonatal AIS, 9 children had presumed AIS and 18 children had presumed PVI. The subjects were 6–18 years old at the time of follow up MRI and did not require anesthesia. One 4.9-year-old subject, with only clinical MRI done under anesthesia was also included.

The demographics of a total of 76 subjects, including 34 stroke patients and 42 healthy controls, is shown in Table 1. There were no differences in sex, mean age at outcome evaluation or the time of MRI investigation (Table 1) between the control, AIS and PVI groups.

**Table 1:** Demographics of the study groups

Demographics	AIS (***n*** = 16)	PVI (***n*** = 18)	Control (***n*** = 42)	***P***-value
Sex Boys/girls	8/8	7/11	24/18	0.429 #
Side of stroke (MRI) left; n (%)	12 (75%)	12 (66.7%)		0.715 *
Age (years) at MRI; mean (95% CI), [range]	11.39 (9.47–13.31) [4.86–17.15]	10.38 (9.04–11.73) [6.78–15.86]	11.59 (10.79–12.39) [8.07–17.86]	0.253 $
AIS classification				
Proximal M1 of the MCA; n (%)	4 (25%)			
Distal M1 of the MCA; n (%)	5 (31.25%)			
Anterior trunk; n (%)	3 (18.75%)			
Posterior trunk; n (%)	4 (25%)			
Age (years) at AHA; mean (95% CI), [range]	8.47 (6.93–10.00) [4.6–13.2]	7.45 (6.18–8.73) [3.5–12.7]		0.208 &
AHA unit; mean (95% CI)	58.56 (48.63–68.50)	64.22 (58.1–70.27)		0.297 §
Right sensorimotor deficit (according to PSOM)				0.272 *
Normal; n (%)	1 (6.3%)	0 (0%)		
Mild; n (%)	4 (25%)	1 (5.6%)		
Moderate; n (%)	4 (25%)	8 (44.4%)		
Severe; n (%)	7 (43.8%)	9 (50.0%)		
PSOM; median (IQR), [range]	2.0 (1.5–2.75) [0.5–7.0]	2.0 (1.0–3.0) [0.5–5.0]		0.713 &
MACS				0.348 *
MACS level I; n (%)	6 (37.5%)	5 (27.8%)		0.545 ‡ #
MACS level II; n(%)	3 (18.8%)	8 (44.4%)		
MACS level III; n (%)	6 (37.5%)	5 (27.8%)		
MACS level IV; n (%)	1 (6.3%)	0 (0%)		

*Abbreviations*: *n* number of patients, *CI* confidence interval, *IQR* interquartile range

^# Chi-square; * Fisher’s exact; $ ANOVA; & Wilcoxon-Mann-Whitney; § t-test;^ ‡ ^Reference group: patients with MACS levels II, III or IV^

The median of the PSOM values for the AIS and PVI groups was not statistically different (*p* = 0.7) and was 2.0 for both groups.

### Hand function according to AHA and MACS

Sixty-nine percent of the patients with AIS (11/16) and 97% (17/18) of the patients with PVI had unilateral spastic CP which was not statistically different between the two groups (*p* = 0.27) (Table 1). Both study groups showed poor affected hand function according to AHA. The mean AHA units were not statistically different in the AIS group compared to the PVI group (58.56 and 64.22, respectively, *p* = 0.3) (Table 1).

The distribution of the patients between AIS and PVI according to MACS did not show a significant difference (Table 1). Of the patients 37% with AIS and 28% with PVI had minor difficulties in handling age-appropriate objects (level I) (*p* = 0.545), while the rest of the patients were classified to have more severely impaired manual ability (levels II-IV) (Table 1).

### Voxel based Morphometry

**Fig. 1:**
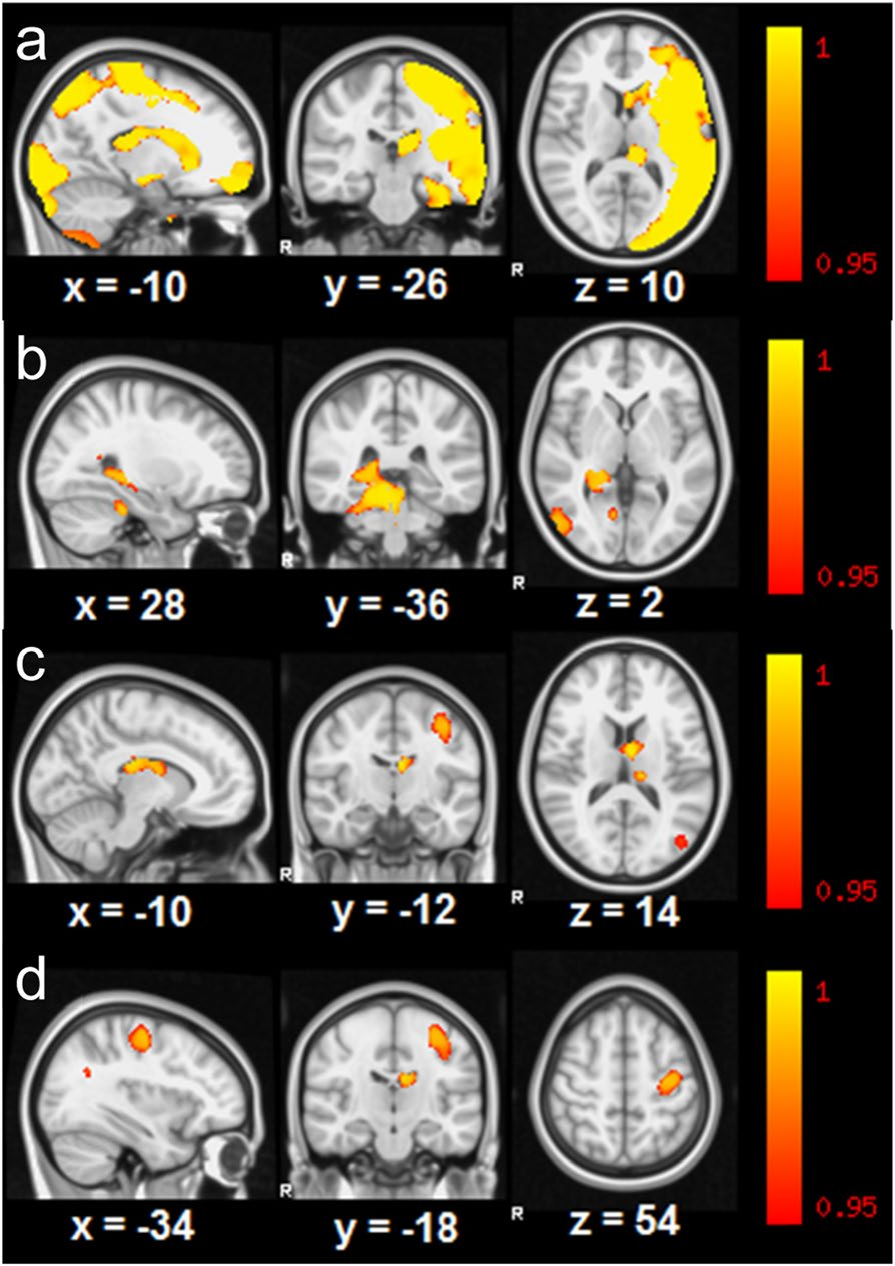
The results of FSLVBM (voxel based morphometry) revealing significant differences in gray matter volume Control>AIS (**a**), AIS > Control (**b**), Control>PVI (**c**, **d**). All images show tfce (threshold-free cluster enhancement) and FWE (family-wise error) corrected t-stats (1-p) > 0.95 with MNI coordinates indicated. Differences in the thalamus and nucleus caudatus for both AIS and PVI (**a**, **c**, **d**); putamen and cortex in (**a**) and motor cortex in (**c**) are presented

Differences in gray matter localization between the AIS, PVI and control groups, calculated by using VBM for the most informative slices, is presented in Fig. 1.

Compared to control, the amount of ipsilesional gray matter in the AIS group was smaller in the cortex corresponding to the lesion and in the nucleus caudatus, putamen, globus pallidus, thalamus, hippocampus and amygdala (Fig. 1a), whereas the amount of gray matter was greater in the contralesional cerebral cortex, thalamus and hippocampus, compared to control (Fig. 1b).

The gray matter volume was only smaller in the ipsilateral nucleus caudatus, in both thalami (Fig. 1c) and in the ipsilesional motor cortex (Fig. 1d) in children with PVI compared to the controls. No contralesional cortical or subcortical regions, exept for the contralesional thalamus, showed statistically significantly smaller gray matter volume in children with PVI compared to controls. Part of the contralesional occipital lobe showed significantly more gray matter in children with PVI compared to controls. However, no equivalent statistically significant difference was found in the subcortical regions.

Children with AIS had less gray matter in the subcortical ipsilateral structures (putamen, hippocampus, amygdala), compared to children with PVI. No region showed statistically more gray matter in AIS compared to PVI.

### Volumetric analysis by segmentation

The normalized mean and median volumes (mm^3^) of the subcortical structures are presented in Table 2. Although the mean age of the children in the study groups was not significantly different (Table 1), the scaling factors were significantly higher for AIS (*p* = 0.005) and PVI (*p* = 0.0001), compared to the control group, but did not differ between AIS and PVI, indicating smaller global brain size in children with PS compared to the control group (Table 2).

**Table 2:** Volumes of normalized subcortical brain structures with between-groups comparisons

Structure normalized [mm3]	Control n = 42	AIS n = 16	PVI n = 18	Control-AIS	Control-PVI	AIS-PVI
Scaling factor for head size; mean (95% CI)	1.38 (1.34–1.41)	1.47 (1.40–1.55)	1.51 (1.45–1.57)	**0.005**	**< 0.0001**	0.369
Range of scaling factor	1.15–1.62	1.26–1.69	1.32–1.69			
Thalamus, ipsilesional; median (IQR)^a^	11,548 (10930–11,948)	9154 (5487–10,017)	9177 (8355–10,069)	**< 0.0001**	**< 0.0001**	0.617
Thalamus, contralesional; mean (95% CI)	11,009 (10787–11,230)	10,312 (9872–10,752)	10,676 (10257–11,095)	**0.003**	0.128	0.171
Caudate nucleus, ipsilesional; mean (95% CI)	5694 (5499–5889)	4905 (4083–5727)	4768 (4283–5252)	0.064	**0.001**	0.756
Caudate nucleus, contralesional; mean (95% CI)	5861 (5684–6037)	5915 (5433–6397)	5770 (5416–6123)	0.787	0.638	0.537
Putamen, ipsilesional; median (IQR)^a^	7355 (6979–7911)	6332 (4198–7756)	7103 (5981–7496)	0.048	0.083	0.523
Putamen, contralesional; mean (95% CI)	7326 (7130–7522)	7851 (7355–8348)	7217 (6889–7544)	0.050	0.545	0.027
Globus pallidus, ipsilesional; median (IQR)^a^	2443 (2331–2535)	2138 (1646–2346)	2231 (2099–2356)	**< 0.001**	**< 0.001**	0.277
Globus pallidus, contralesional; mean (95% CI)	2501 (2443–2558)	2465 (2318–2612)	2540 (2397–2683)	0.633	0.600	0.444
Hippocampus, ipsilesional; median (IQR)^a^	5265 (4994–5451)	4422 (3875–5049)	4862 (4540–5283)	**0.004**	**0.031**	0.125
Hippocampus, contralesional; mean (95% CI)	5293 (5141–5445)	5379 (5044–5714)	5210 (4933–5486)	0.586	0.583	0.361
Amygdala, ipsilesional, mean (95% CI)	1610 (1504–1716)	1421 (1212–1629)	1537 (1377–1697)	0.068	0.458	0.334
Amygdala, contralesional; mean (95% CI)	1540 (1454–1626)	1554 (1365–1743)	1562 (1416–1709)	0.877	0.796	0.936
Nucleus accumbens, ipsilesional; mean (95% CI)	756 (711–801)	524 (416–633)	701 (601–802)	**< 0.0001**	0.268	**0.004**
Nucleus accumbens, contralesional; median (IQR)^a^	593 (534–692)	631 (477–705)	611 (523–686)	0.924	0.790	0.904

Subcortical brain structures segmented using the FSL’s FIRST tool and the volumes normalized by the FSL’s SIENAX tool’s volumetric scaling factor in mm3. *Abbreviations*: *CI* Confidence interval, *IQR* interquartile range. Between-groups comparison, using ANOVA or Kruskal-Wallis test (in absence of normal distribution denoted by with ^a^). After FDR correction, using level 0.05, the cutoff *p-*value for significance of a single comparison was 0.033

The ipsi- and contralesional thalamus, ipsilesional globus pallidus, hippocampus and nucleus accumbens had a significantly smaller volume in the AIS group compared to healthy controls (Table 2).

Like the AIS group, the PVI group showed a significantly smaller volume of the ipsilesional caudate nucleus, globus pallidus, thalamus and hippocampus, compared to healthy controls. In PVI, the contralesional subcortical structures showed no significant difference compared to control (Table 2).

### Correlations between volumetric analysis by segmentation and affected hand function according to AHA

**Fig. 2:**
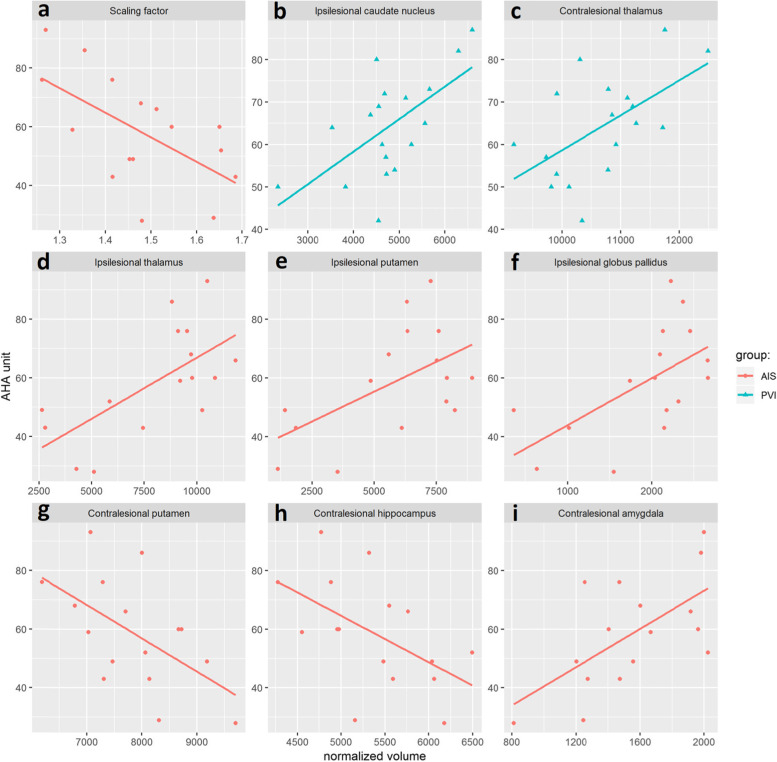
Statistically significant linear correlations between affected hand function, based on AHA test, and volume of the subcortical structures for children with AIS and PVI. (**a**) subject’s head size measured by using FSL’s SIENAX tool’s volumetric scaling factor, (**b**) ipsilesional caudate nucleus, (**c**) contralesional thalamus, (**d**) ipsilesional thalamus, (**e**) ipsilesional putamen, (**f**) Ipsilesional globus pallidus, (**g**) contralesional putamen, (**h**) contralesional hippocampus, (**i**) contralesional amygdala

Pearson’s and Spearman’s rank correlation between AHA units and normalized subcortical volumes are presented in Table 3. Figure 2 illustrates the graphic representation of the individual data points and of the linear regression line for the structures for which it was significant and valid to use a linear model.

**Table 3:** Correlation of AHA units with normalized subcortical brain structure

Structure	AIS *n* = 16		PVI *n* = 18	
	r	p	r	p
Scaling factor for head size	−0.60	**0.013**	−0.30	0.231
Thalamus, ipsilesional	0.66	**0.006**	0.83 ^a^	**<.0001**
Thalamus, contralesional	−0.11	0.680	0.57	**0.013**
Caudate nucleus, ipsilesional	0.35	0.179	0.62	**0.007**
Caudate nucleus, contralesional	−0.47	0.065	−0.01	0.958
Putamen, ipsilesional	0.55	**0.026**	0.20	0.428
Putamen, contralesional	−0.57	**0.022**	−0.25	0.310
Globus pallidus, ipsilesional	0.59	**0.016**	0.46 ^a^	0.053
Globus pallidus, contralesional	0.09	0.748	0.09	0.709
Hippocampus, ipsilesional	0.51 ^a^	**0.044**	0.16	0.527
Hippocampus, contralesional	−0.53	**0.033**	−0.01	0.981
Amygdala, ipsilesional	0.59 ^a^	**0.015**	0.30	0.230
Amygdala, contralesional	0.62	**0.010**	−0.14	0.579
Nucleus accumbens, ipsilesional	0.41	0.111	0.18	0.463
Nucleus accumbens, contralesional	0.42	0.104	−0.17	0.496

Volumes normalized by head size using the FSL’s SIENAX tool volumetric scaling factor. Pearson’s correlation coefficient is provided in case the assumptions of the linear regression model are satisfied. When these assumptions are not satisfied, Spearman’s rank correlation coefficient is provided and noted by ^a^. After FDR correction, using level 0.08, the cutoff *p*-value for significance of a single comparison was 0.048 (for the AIS group) and 0.016 (for the PVI group) marked in bold

In contrast to children with PVI, for children with AIS smaller head size (based on the higher subcortical volumetric scaling factor) correlated with worse affected hand motor function in AHA units (Table 3, Fig. 2a).

In children with AIS, larger size of the ipsilesional putamen, globus pallidum, thalamus, hippocampus, amygdala and contralesional amygdala was correlated with better bimanual motor outcome (higher AHA units) (Fig. 2e, f, d, I; Table 3) and larger size of contralesional putamen and hippocampus was correlated with worse bimanual motor outcome (lower AHA units) (Fig. 2g, h; Table 3).

In children with PVI, larger volume of the ipsilesional thalamus, nucleus caudatus and contralesional thalamus was correlated with better bimanual motor outcome (higher AHA units) (Fig. 2b, c; Table 3).

### Correlations between volumetric analysis by segmentation and bimanual hand function according to MACS

The mean normalized volumes of the subcortical structures in relation to bimanual hand function measured in MACS levels did not differ significantly between children with AIS and PVI (Table 4).

**Table 4:** Comparison of normalized subcortical brain structure volumes in mm^3^ in AIS and PVI children at different bimanual motor outcome levels of the MACS scale

Structure normalized [mm3]	AIS *n* = 16				PVI *n* = 18			
MACS	I *n* = 6	II *n* = 3	III-IV *n* = 7	*p*-value	I *n* = 5	II *n* = 8	III-IV n = 5	*p* value
	mean (95% CI) or median (IQR)*	mean (95% CI) or median (IQR)*	mean (95% CI) or median (IQR)*		mean (95% CI)	mean (95% CI)	mean (95% CI)	
Thalamus, ipsilesional	9919 (8758–11,080)	9945 (7865–12,025)	5485 (2991–7980)	**0.003 #$**	10,649 (8721–12,577)	9162 (8578–9746)	6895 (4187–9603)	**0.004 #$**
Thalamus, contralesional	10,110 (9643–10,307)*	10,237 (10227–11,289)*	10,291 (9587–10,988)*	0.861 *	11,371 (10364–12,378)	10,544 (9830–11,258)	10,190 (9709–10,672)	0.062
Caudate nucleus, ipsilesional	5266 (4345–6186)	5872 (2560–9183)	4182 (2449–5915)	0.355	5416 (4205–6628)	4801 (4218–5383)	4067 (2769–5364)	0.082
Caudate nucleus, contralesional	5506 (4761–6252)	5987 (4539–7435)	6235 (5211–7258)	0.372	5664 (4678–6649)	5825 (5178–6472)	5787 (4954–6620)	0.931
Putamen, ipsilesional	6778 (5926–7629)	7242 (1967–12,518)	4314 (1469–7159)	0.248	7099 (5677–8522)	6984 (6344–7624)	6505 (5156–7855)	0.590
Putamen, contralesional	7171 (6486–7856)	8140 (5749–10,530)	8311 (7508–9114)	0.063	7007 (5952–8063)	7185 (6764–7605)	7476 (6569–8384)	0.550
Globus pallidus, ipsilesional	2324 (2097–2551)	2148 (974–3322)	1459 (723–2194)	0.122	2415 (2052–2778)	2236 (2107–2365)	1953 (1456–2451)	0.055
Globus pallidus, contralesional	2469 (2209–2730)	2606 (1657–3556)	2400 (2147–2653)	0.588	2575 (2145–3005)	2604 (2373–2834)	2403 (2082–2724)	0.478
Hippocampus, ipsilesional	4831 (4252–5410)	4911 (3044–6778)	3881 (2874–4888)	0.120	4680 (3787–5573)	4960 (4631–5288)	4619 (3518–5720)	0.604
Hippocampus, contralesional	5095 (4516–5673)	4828 (4233–5424)	5858 (5431–6286)	0.010	5140 (4560–5720)	5193 (4652–5734)	5306 (4573–6038)	0.901
Amygdala, ipsilesional	1647 (1312–1983)	1474 (1178–1770)	1203 (805–1602)	0.116	1567 (1229–1904)	1602 (1307–1898)	1402 (981–1824)	0.565
Amygdala, contralesional	1705 (1381–2030)	1679 (985–2374)	1371 (1025–1716)	0.196	1605 (1135–2074)	1531 (1264–1799)	1569 (1317–1821)	0.918
Nucleus accumbens, ipsilesional	632 (439–825)	584 (61–1108)	407 (245–568)	0.110	664 (451–877)	786 (675–897)	603 (243–963)	0.306
Nucleus accumbens, contralesional	630 (501–759)	703 (613–793)	500 (349–651)	0.093	572 (428–717)	655 (534–777)	601 (544–658)	0.458

bold text = significant *p*-value after FDR correction (using level 0.05, the single comparison cutoff *p*-value being 0.0042 for the AIS and PVI groups);

# = mean volume difference between subjects with MACS I and III-IV;

$ = mean volume difference between subjects with MACS II and III-IV;

* = absence of normal distribution (Kruskal-Wallis test used instead of ANOVA)

In children with both AIS and PVI, significant differences were seen in the mean volumes of the ipsilesional thalamus for the different MACS levels (Table 4), while for mean volumes of the other structures the differences were not significant (Table 4). Children with AIS with more severely affected bimanual hand function (MACS III-IV) had reduced mean ipsilesional thalamus volume compared to children with less severe hemiplegia (MACS level I (*p* = 0.002) and MACS level II (*p* = 0.006)). Among children with PVI, a significantly smaller mean volume of the ipsilesional thalamus was associated with severe hemiplegia (MACS levels III-IV) as compared to those with less severe hemiplegia (MACS level I (*p* = 0.001) and MACS level II (*p* = 0.016)).

## Discussion

The current study identified widespread differences in the volume of the thalamus, basal ganglia and hippocampus in children with unilateral spastic CP following ischemic PS, compared to controls. Reduction in the size of the ipsilesional thalamus was significantly associated with worse function of the affected hand in both stroke subgroups. The two study groups, AIS and PVI, showed different patterns for correlations between hand function and volume of the other subcortical structures.

In children with AIS, poor affected hand function was correlated with smaller volume of the thalamus, putamen, globus pallidus, amygdala and hippocampus. In children with PVI smaller size of the thalami and ipsilesional nucleus caudatus was correlated with poor hand function.

There are differences in the patterns of damage to the basal ganglia and thalamus in different vascular types of PS. Presumed PVI, evolving after germinal matrix hemorrhage before 34 weeks of gestation during the antenatal period, leads to venous thrombosis and brain damage in the periventricular area including likely also the nucleus caudatus. Neonatal and presumed perinatal AIS arise around term birth, when the child’s vascular structures are already well developed. Occlusions of the middle cerebral artery, including the lenticulostriate arteries, cause different patterns of damage depending on the site of the thrombus [5, 7–9]. An earlier study of childhood AIS has also reported diaschisis of different brain regions after stroke, based on diffusion-weighted imaging, which carries an important clinical implication for both cognitive and motor outcomes in these children [30]. Another study of neonatal AIS children with severe contralesional hand motor deficit exhibited significant volume reduction in the thalamus [32].

In recent years, involvement of the subcortical structures and their importance in predicting hemiplegia following ischemic PS has received increased attention. It has been shown that relative stroke volume and involvement of the basal ganglia in acute phase imaging significantly predicts the diagnosis of CP at 2 years of age [13]. While direct damage to the thalamus does not occur in the case of typical infarctions, their high connectivity to injured areas may result in structural and functional alterations. Consistent with previous results [12], our study shows that ipsilesional thalamic volume loss is not limited to large cortical strokes typical of AIS, but occurs also after smaller periventricular white matter injuries.

In our AIS group, significant ipsilesional volume changes were found, besides the thalamus also, in the hippocampus and in parts of the basal ganglia. Additionally, the volume loss of the caudate nucleus, putamen and amygdala was close to being statistically significant. In contrast to the study of Craig and colleagues [12], our study found significant correlations between volume loss of the ipsilesional subcortical structures and thalamus and severity of affected hand function. These results are in line with a previous study of AIS children focusing on contralesional gross manual dexterity assessed by the box and block test and whole brain gray and white matter volume changes on high-resolution magnetic resonance imaging [32]. In that study contralesional hand performance after neonatal AIS was correlated with smaller volume of the ipsilesional mediodorsal thalamus.

Craig et al. [12] reported larger volumes of the contralesional thalamus in patients with AIS versus control and PVI. In their opinion a larger contralesional thalamus does not imply that it succesfully takes over the function, but is probably related to poor outcome [12]. In our study, reduced volume of the contralesional thalamus in children with AIS versus control did not correlate with hand function. In contrast, use of the VBM method revealed increased gray matter volume in part of the contralesional thalamus compared to control. This finding can be explained by the fact that VBM distinguishes changes in different locations of the same structure, while segmentation summarizes the volume of the entire structure. In children with PVI, volume differences in the contralateral thalamus were not significant and larger contralesional thalamus was correlated with better hand function. One reason for the discrepancy between our results and those of Craig et al. [12] might be the differences in the patient inclusion criteria, as their sample was restricted to relatively well–functioning children. This selection bias may have rendered their results less representative for the most severely affected children, as noted also by the authors [12]. Another explanation is that we analyzed correlations between volume of the basal ganglia and thalamus separately in AIS and PVI children, whereas the above study analyzed PVI and AIS combined as one single group. In our study different correlations occurred between volume of the contralesional thalamus and hand motor function for the PVI and AIS groups.

We found significant correlations of larger contralesional putamen and hippocampus with poor hand function in AIS, demonstrating that larger volumes of contralesional structures such as the putamen and hippocampus may indicate worse motor performance. Further studies with large groups are needed to evaluate the discrepancy between the results of contralesional volume changes in the thalamus and basal ganglia.

In our study, the volume difference in PVI patients compared to controls was limited to the ipsilesional thalamus, nucleus caudatus, globus pallidus and hippocampus. According to earlier studies, the cortex is relatively well spared in children with PVI [7, 46]. However, in our subjects the ipsilesional cortical motor area was also smaller, indicating diaschisis in this area, which may provide less input to the basal ganglia, leading to their smaller volume outside primary white matter damage. Resting state functional MRI has shown correlation between better functional connectivity and worse hand function in children with PVI [46]. However, that study only evaluated the functional connectivity of the sensomotor cortex and thalamus without including the other basal ganglia in the analysis. Our findings demonstrate once more that the brain is an immense network and a damage that causes changes in brain structures distant from the damaged area leads also to changes in function. Thus, further analysis is needed to evaluate the functional connectivity of the basal ganglia in motor outcome studies of PS.

Our study shows also volume differences in the ipsilesional hippocampus in children with AIS but not PVI, compared to controls, which can be explained by differences in the size and location of vascular damage. As correlations of volume of the contralesional hippocampus and amygdala with motor function are difficult to explain, further research should also deal with more comorbidities like, e.g. epilepsy.

Several significant limitations of the study should be considered. Our study group consisted of children with a wide age range, which introduces variance regarding brain volumes and outcomes. We attempted to correct it by normalizing brain volumes. Also some subjects underwent MRI for the current study with a time lag from clinical evaluations. It is unknown how the affected hand function in individual children might have changed or might have been affected by interventions and therapies during this delay. However, only a few children with PS received regular rehabilitation. It has been shown that during development, children with hemiplegic CP tend to acquire greater skill with the unaffected hand and increasingly neglect the impaired hand. The function of the affected hand reaches a plateau between 2.5 and 8 years of age and does not change significantly thereafter [47].

We applied two methods, VBM and volumetric analysis by segmentation, for analyzing the volume of the subcortical structures in children with PS. VBM has the advantage in being more automated than manual segmentation and in providing information about the entire brain. However its execution as well as interpretation of results are challenging because of brain damage after PS and require more experience from the person who performs measurements. Segmentation of a lesioned brain with the use of the FSL FIRST tool is only partially automated and volumetry is more time consuming and limited to the subcortical structures only. However, the results obtained with either method revealed widespread volume differences, compared to control, in the ipsi- and contralesional basal ganglia, thalami and hippocampi, which correlated with hand motor function both in AIS and PVI.

Intrarater and interrater reliability of segmentation corrections was not assessed, as segmentation was automatically performed for all subjects, while additonal manual corrections were made for subjects with severe lesions who had major displacements and atrophy of the subcortical structures. Still, all subjects were evaluated in a random order by two investigators and re/evaluated after 2 weeks to minimize any bias.

Althoug there were more girls in the PVI group than in the AIS group, the difference was not significant. This reflects the natural occurence of the disease with a higher number of girls among PVI patients also in our previously studied cohort of PSD [5], compared to a normally slightly higher number of boys in AIS studies [48, 49]. Like previous studies of thalamic volume loss [12] and contralesional cerebellum volume loss [31], our study included only a small number of the most severely injured patients with proximal M1 stroke with direct involvement of the basal ganglia. It is noteworthy that in our study the basal ganglia and thalami were affected on a larger scale even in children with more peripheral vascular involvement.

As reported earlier [12], evaluation of the volume of the basal ganglia and thalamus as the key components of the sensorimotor network alongside with the cortical structures, corticospinal tracts and cerebellum, might add prognostic information about both vascular types of ischemic PS, especially in children who lack acute phase images, or whose images do not reveal direct injury to these structures.

## Conclusions

PS causes widespread volumetric changes in the ipsi- and contralesional subcortical structures with varying patterns in AIS and PVI. Smaller volume of ipsilesional thalamus was associated with poor affected hand function regardless of the PS subtype. The pattern of correlation between hand function and volume of the other subcortical structures varies between children with PVI and AIS. The size of the nucleus caudatus is affected the most in children with PVI and the size of the putamen, globus pallidus, amygdala and hippocampus, in children with AIS. Radiologic evaluation of subcortical structures could add to the understanding of developmental neuroplasticity following perinatal stroke and is important in predicting outcomes, as well as in development of individualized therapies.

## Data Availability

The datasets generated and/or analysed during the current study are not publicly available due to requirement for patient confidentiality, but are available from the corresponding author on reasonable request.

## Abbreviations

AHA: Assisting Hand Assessment
AIS: Arterial ischemic stroke
ANOVA: Analysis of variance
CI: Confidence interval
CP: Cerebral Palsy
FDR: False discovery rate
FSL: FMRIB Software Library
FWE: Family-wise error
IQR: Interquartile range
MACS: Manual Ability Classification System
MCA: Middle cerebral artery
MRI: Magnetic resonance imaging
PLIC: Posterior limb of the internal capsule
PS: Perinatal stroke
PSD: Pediatric Stroke Database
PSOM: Pediatric Stroke Outcome Measure
PVI: Periventricular venous infarction
VBM: Voxel based morphometry
